# Neanderthals as familiar strangers and the human spark: How the ‘golden years’ of Neanderthal research reopen the question of human uniqueness

**DOI:** 10.1007/s40656-020-00327-w

**Published:** 2020-07-21

**Authors:** Susan Peeters, Hub Zwart

**Affiliations:** 1 Institute for Science in Society (ISiS), Radboud University Nijmegen, Huygens building - Heyendaalseweg 135, 6525 AJ Nijmegen, The Netherlands; 2 Erasmus School of Philosophy (ESPhil), Erasmus University Rotterdam, Bayle building - Burgemeester Oudlaan 50, 3062 PA Rotterdam, The Netherlands

**Keywords:** Neanderthal research, Paleogenomics, Val Plumwood, Palaeoanthropology, Anthropocentrism, Philosophical anthropology

## Abstract

During the past decades, our image of *Homo neanderthalensis* has changed dramatically. Initially, Neanderthals were seen as primitive brutes. Increasingly, however, Neanderthals are regarded as basically human. New discoveries and technologies have led to an avalanche of data, and as a result of that it becomes increasingly difficult to pinpoint what the difference between modern humans and Neanderthals really is. And yet, the persistent quest for a minimal difference which separates them from us is still noticeable in Neanderthal research. Neanderthal discourse is a vantage point from which the logic of ‘us’ versus ‘other’ is critically reconsidered. Studying contemporary academic literature and science autobiographies from an oblique perspective, focusing not on Neanderthals as objects, but on the dynamics of interaction between Neanderthal researchers and their finds, basic convictions at work in this type of research are retrieved. What is at issue is not the actual distinction between modern humans and Neanderthals (which is continuously being redefined), but rather the dualistic construction of human and nonhuman. Neanderthal understanding is affected by the desire to safeguard human uniqueness. The overall trend is to identify the human mark or spark, which defines us as favoured ‘winners’. The paradoxes emerging in contemporary Neanderthal discourse are symptomatic of the fact that a dualistic style of thinking is no longer tenable.

## Introduction

In every-day language, the label ‘Neanderthal’ has become synonymous with sluggish, brutish cave dwellers, primitive pre-humans who were decidedly uncivilized, unintelligent and uncouth. Moreover, until recently, as we will demonstrate in this article, scientific discourse basically seemed to confirm this wide-spread stereotypical view. To most paleoanthropologists, they simply did not ‘cut the mustard’ as humans (Speth 2004) and were considered inferior to modern humans in a wide range of domains. At the moment, however, this image of Neanderthal is changing rapidly and drastically, in the academic literature, but also in the public domain, and a growing sense of ‘collective guilt’ even seems noticeable concerning our pervasive and prejudiced underestimation in the past (Papagianni and Morse 2015). In 2017, Neanderthals made the cover of *The New York Times Magazine*, featuring an article on the ways scientific perceptions of Neanderthals have changed over time, attempting to show how ‘we’ denied for so long that ‘they’ were people too (Mooallem 2017). The cover implied that we were in fact the stupid ones, for getting Neanderthals so wrong. In an exhibition about Neanderthals in the Maas Valley in the Netherlands (2015), the picture was even reversed. Compared to ourselves, the strong and intelligent Neanderthals, who were able to survive several ice ages, must have been ‘super people’. The overall trend in the academic literature is to see Neanderthals increasingly as neither subhuman nor superhuman, but as basically human. In a recent comprehensive review of Neanderthal research, archaeologist Papagianni and science historian Morse describe how modern science, enabled by the latest research technologies, is drastically rewriting the story of the Neanderthals (Papagianni and Morse 2015). In this ‘golden age of Neanderthal research’ (p. 14), a mounting body of evidence continues to expand the known repertoire of sophisticated strategies and symbolism practiced by Neanderthals, and sapiens-centrism has come under pressure. The more data we gather on their behaviour, the more similar Neanderthals seem to be to the modern human pattern. Not only dental hygiene, also large-scale cooperative hunting, complex stone tools, language, planning, care for the ill, imagination and symbolic behaviour, was present in Neanderthals. The only traceable advantage of *Homo sapiens* was that they had started to produce ornaments with little beads and shells, something which seemed absent in Neanderthal culture (p. 21). Recent research, however, yielded perforated and ochre marine shells and colorants attributed to Neanderthals, suggesting once again that they were cognitively indistinguishable from modern humans (Hoffmann et al. 2018). On the other hand, the authors still describe Neanderthals as anatomically ‘unmistakably different’ (p. 12). Resolving the tension between these two apparently incompatible positions (same, yet different) is central to understanding what it means to be human, the authors state (p. 58).

By being a human species (belonging to the genus *Homo*), and yet ‘not-us’ (as *Homo sapiens*) at the same time, Neanderthals are especially fascinating. The genus *Homo* is a messy, contested category that is closely tied to the concept of being human, and the boundaries of both the species and the genus remain fuzzy (Collard and Wood 2015; Schwartz 2016; Wood and Collard 1999). The genus was established in 1735 by Carolus Linnaeus as part of the classification system he introduced in his Systema Naturae. Instead of any specific identifying characteristics, he described *Homo* with the words *nosce te ipsum* (know thyself). After the discovery of the partial skeleton in Neander Valley in 1856, William King introduced the species name *Homo neanderthalensis*, for the first time expanding our genus beyond *Homo sapiens* (King 1864). For King, the brain size suggested it was human enough to belong to the human genus, but the ape-like brain shape indicated that there was a wide gap separating the Neanderthal—characterized by a similar darkness such as that experienced by the chimpanzee (p. 96)—from the human species. To most researchers however, the Neanderthal represented an ancient, inferior race of *Homo sapiens*, an extension into the past of the hierarchy of living human ‘races’, descending from civilized to savages (Spencer 1984). By the end of the century, with the discovery of more Neanderthal fossils and the even more ancient *Pithecanthropus*, Neanderthals were generally accepted as an extinct species of human, either ancestral to modern humans, or an evolutionary dead-end. Although Neanderthals have moved to-and-fro between animality and humanness (Corbey 2005), and into and out of the direct lineage of human ancestry, the prevailing view considered them to be a crude prototype of our own species (for a historical overview see for example Drell 2000; Goodrum 2016; Hammond 1982; Madison 2016; Sommer 2006; Spencer 1984; Trinkaus and Shipman 1993; Zilhão 2001).

Neanderthals are our closest relatives and the best-known and longest known hominin^1^ species, other than ourselves. They lived in Europe for hundreds of thousands of years and coexisted with modern humans until approximately 40.000 years ago. According to some, Neanderthals are unquestionably the best mirror that we can hold up to ourselves, and therefore of singular importance to help us discover wherein our uniqueness lies (Tattersall 2002). Others see them as an exemplification of alternative ways of humanness (Holden 1998). Palaeoanthropology as a distinct discipline began with the discovery of the Neanderthal skeleton in 1856 (Stringer and Gamble 1993). Right from the very beginning, the research field became notorious for its intense rivalries and fierce controversies, which often go beyond the more usual forms of academic disagreement (Lewin 1997). One of the reasons may be that paleoanthropological research touches on some of the most basic and sensitive questions that humans ask themselves; who are we, where do we come from, and what is our place in the world? Like primatology, which according to Donna Haraway is ‘about the construction of self from the raw material of other’ (Haraway 1989), palaeoanthropology is concerned with the identity of our own species. Paleoanthropologists are policing and at the same time questioning the boundary between humans and nonhumans, thereby defining and redefining what it means to be human (Cartmill 1990; Roebroeks 1995). In this negotiation of human identity, in the boundary zone between human and animal, modern and archaic, self and other, the other often serves as a foil, a caricature even, which indirectly reveals what we apparently consider as ‘us’. Proximate others, close to us, yet different, are boundary creatures which threaten the stability of human identity by blurring and challenging some firmly established categories. Neanderthals are at the centre of one of the hottest debates in palaeoanthropology, and such controversies mostly revolve around issues of identity and equality (Graves 1991). Are they like us? The interest in Neanderthals is therefore also an interest in ourselves; raising the question where to draw the line. To paraphrase Haraway: this explains both the importance and the controversial nature of Neanderthal visions.^2^

Neanderthal research has clearly progressed, due to new fossil discoveries, improved methods for dating archaeological evidence and CT-scanning, and developments in the study of ancient DNA (paleogenomics) and ancient proteins (paleoproteomics), for example. This allowed researchers to generate and assess an avalanche of data, shedding more light on Neanderthal life, changing how we see them, but also challenging the way we see ourselves. And this happens at a crucial moment when we are already questioning in a very fundamental manner a number of basic concepts such as nature, technology and the human, due to the fact that, at present, during the Anthropocene^3^ (the era of humankind) we are destroying our habitat and facing a global environmental crisis. Against this backdrop, questions such as ‘who are we?’ and ‘what is our place in the world?’ are becoming increasingly urgent, and the standard answers increasingly unconvincing. We need to thoroughly rethink human beings, question some very basic assumptions, and in such a setting Neanderthal research raises more questions than merely paleoanthropological ones.

Recent genetic evidence and archaeological data show that Neanderthals and their modern humans contemporaries were very similar in biological and cultural capacities (Roebroeks and Soressi 2016), and the predominant opinion now appears to be that, yes, Neanderthals were people like us, ‘our equal in humanity’ (Papagianni and Morse 2015: p. 13) Nevertheless, the persistent quest for a minimal difference which separates them from us, for the miraculous spark that made us radically different, continues. What if there is no final answer to the question whether Neanderthals are ‘really’ human or not? What if we no longer consider ‘humanness’ as the measure of all things? Instead of where to draw the line, maybe the right question to ask would be: why do we persist in drawing a line in the first place?

In this article, we argue that this persistent emphasis on difference has to do with the way we have conceptualized ourselves as human beings, and that we should make use of current Neanderthal discourse to re-examine the concept of the human. How is the meaning of humanness constructed and defended? What basic concepts and convictions are, often implicitly, at work in palaeoanthropology? We argue that the traditional concept of humanity is part of an interrelated set of mutually reinforcing dualisms. Building on the work of Val Plumwood and others, we argue that the paradoxes emerging in contemporary Neanderthal discourse are symptomatic of the fact that this dualistic style of thinking is no longer tenable. Neanderthal research is not only interesting in itself, but challenges the status quo, by complicating our self-image and challenging the anthropocentric framework of a dominant intellectual and cultural tradition. More precisely, whereas traditional palaeoanthropology reflects a dualistic, Darwinian logic of winners and losers, of ‘favoured’^4^ and non-favoured races, Neanderthal research forces us to reconsider the tendency to frame early human history in terms of ‘them’ and ‘us’.

## The logic of ‘us’ versus ‘other’

As indicated above, the aim is to understand Neanderthals in terms of their humanity. But deciding what is human is not a purely scientific matter, for it includes normative assumptions about what is to be considered truly human. Historically, moreover, there has always been a tendency to reserve the label ‘human’ to a small subset of (favoured) human beings (Stuurman 2017). In the aftermath of the Second World War, a statement by UNESCO aimed to replace the notion of a hierarchical subordination of human populations with a belief in the unity, continuity and equality of all human beings (Cartmill et al. 1986). More recently, the discussion has been raised whether other species, notably the great apes, should be included in the ‘community of equals’, based on their relatedness and similarity to humans (Cavalieri 2015; Cavalieri and Singer 1993). Palaeoanthropology, as indicated, extends the discussion to our Pleistocene ancestors, notably (but not exclusively) Neanderthals.

Building on the work of Plumwood as a conceptual framework, we will argue that merely expanding the scope of what counts as human means redefining the boundaries along slightly different lines of inclusion and exclusion, while the yardstick of inclusion (namely: similarity to those deemed to be fully human) remains unchallenged. Numerous anatomical and behavioural features have been used as markers of humanness, such as upright bipedalism, manual dexterity, certain facial or cranial angles, cranial capacity, language, the extent of rational control over emotional impulse, conceptual thinking, art, self-recognition and tool making. Much scientific effort and ingenuity has gone into redefining these criteria as soon as nonhumans seemed to qualify as well, re-establishing the alleged human uniqueness (Cartmill 1990; Corbey 2005). What is at issue, we argue, is not where to draw the line, but why we should want to draw a line in the first place. This practice of line-drawing as such should be regarded as a symptom of anthropocentrism, or human-centeredness, which not only means placing the human in the centre of beings, but also entails the desire to determine human specificity over and against those beings who/that threaten that specificity (Calarco 2008, 2015). What is at issue is not whether Neanderthals should be defined in terms of difference or sameness, but rather the dualistic construction of human and nonhuman as such. We will be unable to understand Neanderthals in terms of their humanity as long as our conceptualisation of humanity is grounded in the desire to find a defining difference between us and them.

Philosopher and ecofeminist Val Plumwood is known for her critical assessment of anthropocentrism, which she describes as a value system that rests on the assumption of a deep dividing line which separates humanity from nature. Plumwood is not the first to address this division, but in her book *Feminism and the Mastery of Nature* (1993a), she develops a comprehensive systematic analysis of how anthropocentrism has been constructed and how it informs many of the core categories of western thought. Plumwood argues that the concept of the human is highly problematic because it is based on a framework of exclusion, denial and denigration of the nonhuman. The characteristics of the genuinely human are polarized and defined in opposition to what is taken to be ‘the natural’—and this includes those humans who are still regarded as being embedded in nature. She draws on feminist theory, arguing that the other is seen, not only as radically separate, but also as inferior, notably in the sense that the other’s agency is denied or minimized, thereby providing a natural background for defining the modern human self. This human-nature distinction has a gendered character, as the concept of humanity is tightly linked to rationality and masculinity. While nature includes everything that reason excludes, this dualistic system of thinking creates value hierarchies that systematically renders inferior everything associated with nature rather than reason, e.g. women, indigenous people, early human ancestors and the other-than-human world.

According to Plumwood, the leading dualisms in western culture are human/nature, mental/manual, civilized/primitive and male/female. A dualism is ‘a relation of separation and domination inscribed and naturalized in culture and characterized by radical exclusion’ (p. 47). Binarizing is a necessary constituent of the process of dualizing, but not the only one. Dualizing also involves polarization and polarization and hierarchy: introducing distance and opposition between elements which are constructed as higher and lower, superior and inferior, as ruler and ruled. Thus, entities are regarded not merely as different, but as belonging to radically different orders or kinds, and hence as not open to change. This is what Plumwood terms ‘hyperseparation’: the radical exclusion of the other, seeing differences not as relative, but as differences in kind. While ‘backgrounding’ the other, this strategy denies dependency, continuity and relationship between the self and the other. Another important characteristic of dualism is ‘relational definition’, meaning that one side defines the other. Whatever is essential to defining the one side serves to define the other side as well, for hyperseparation is always a matter of presence or absence, of privilege or lack. The other is objectified and stereotyped. In this way, the other’s independent identity is denied, for the other is primarily seen as not-us or not-quite-us. The domination/subordination relationship determines the identity of both opposites.

This hyperseparated conception of the human expresses a ‘master perspective’ and sees the essentially human as part of a radically separate and higher order of reason, mind or consciousness, set apart from the lower order of naturalness, where agency and intelligence are lacking and which comprises the body, the woman, the animal and the pre-human. It is a hierarchical conceptual system for sorting, organizing and understanding the world around us, an effective way to position ourselves and orient our research practices, providing a symbolical order, but also an intellectual basis for human-centeredness and domination, making it seem inevitable, self-evident and natural. Difference, in this perspective, is not a neutral equation, but convey polarity and hierarchy. According to Plumwood, however, this dualistic perspective can no longer be taken for granted as ‘the’ human model, since this way of thinking has resulted in a massive destruction of the biosphere. It is an egoistic model, a master story of western culture which sees history in terms of conquest and control, appropriation and exploitation, destruction and incorporation.

One area in which the question of what is human is very prominent, is in the relation of humans to animals. Animals present complex interplays of similarity and difference. On the basis of dualistic assumptions, a recognition of others as ‘alike yet different’ seems impossible. They are either valued in human terms (so that their otherness is lost) or constructed as different (suggesting a hierarchical divide between them and us). The more similar to humans an animal is, the more apparent the tension becomes. When genetic similarity between humans and primates is emphasized, for example, separation is safeguarded by producing yet another uniquely human trait, or by constructing difference in terms of the function of these shared genes (Holmberg 2005).

When it comes to determining the relationship between modern humans to other species of humans, dualistic thinking becomes even more problematic. While we regard them as basically human, we still try to distinguish ourselves as human beings and use these proximate others to define and redefine our own self-image. Neanderthals are praised or disqualified in terms of their conformity to a concept of the ‘fully human’, but affirming full humanity can never go without redefining the model, that is grounded in the exclusion of a vast number of beings, deemed nonhuman or not fully human. The positions of ‘being the same as’ or ‘different from’ mark asymmetrical power relations, signifying differences that are organized according to a hierarchical scale. ‘To be different’ therefore implies ‘to be less than’ (Braidotti 2002). Indeed, the view that an alleged superiority of modern humans led to the demise of the Neanderthals, is still a prominent one (Marean 2015; Roebroeks and Soressi 2016). Neanderthal discourse is a vantage point from which we may reconsider the logic of ‘us’ versus ‘other’. It provides a critical window into the web of interconnected, mutually reinforcing dualisms, and allows us to recognize the complex, interacting patterns of continuity and difference. In this way we are enabled to discern the traditional patterns of exclusion and domination, and the anthropocentric logic at work in the formation of different kinds of oppositions and hierarchies.

We will therefore study contemporary (concentrating on the twenty-first century, notably the past decade) Neanderthal discourse from an oblique perspective (Zwart 2017), which focusses not on Neanderthals as ‘object’, but on the dynamics of interaction between Neanderthal researchers and their finds, paying special attention to documented case studies as exemplifications of this dynamics, in order to retrieve the basic convictions (the ‘philosophemes’) at work in this type of research. Rather than on bone fragments, stone artefacts or genes, the focus is on the way in which these findings are presented: on the terminology that is being used, the images that are being projected, the metaphors that are employed, and the controversies or misunderstandings that are evoked. The focus of attention shifts from the object pole to the ways in which ‘subject’ (the Neanderthal expert) and ‘object’ (the Neanderthal world) are intimately involved with one another. Although the *subject* is a modern researcher and the object may be a Neanderthal *bone* from the Pleistocene era, this polarity between subject and object becomes problematic as Neanderthals are coming increasingly closer. One of the big methodological challenges of Neanderthal research is how to prevent genetic contamination of Neanderthal remains with modern DNA (Pääbo 2014a), but a similar methodological challenge is to detect and prevent *epistemic* contamination, i.e. the extent to which our vision of Neanderthal existence becomes inevitably contaminated by our modern preconceptions and desires. Rather than striving for a completely objective portrayal, we should at least be aware of the extent to which such preconceptions affect the ways in which Neanderthal and *Homo sapiens* (‘others’ and ‘ancestors’) are actually envisioned. How are dualisms mobilized to construct differences in terms of inferior and alien?

Paleogenomics plays an important (albeit highly ambiguous) role in this. Improved methods for the extraction of ancient DNA from fossils and paleontological sites and advances in genome sequencing technologies have revolutionized the research on ancient organisms, extinct species, and past environments. At present, whole genomes have been sequenced from numerous ancient individuals and extinct species (Der Sarkissian et al. 2015). DNA is seen as a more definite source of information than traditional tools of archaeology, and paleogenomics as a more data-oriented way to understand what makes humans unique. However, this research field was guided from the very outset by the desire to discover the factor X, i.e. the set of unique human genes that allows researchers to distinguish them from us. At the same time, the actual results of paleogenomics consistently seem to thwart such expectations. In other words, paleogenomics initially seems to endorse but eventually seems to undermine what Plumwood refers to as hyperseparation.

## Paleogenomics and the quest for the factor X

During the 1980s, molecular biology and genomics entered the field of palaeoanthropology. Genetic studies suggested that modern human’s mitochondrial Eve’ lived about 200,000 years ago in Africa (Cann et al. 1987; Wilson et al. 1987). This suggested that archaic human species were replaced by the invading *Homo sapiens* from Africa without any interbreeding. The first studies on Neanderthal DNA in 1997 supported this. When a complete mitochondrial genome sequence was reconstructed, using the original bones found in the Neander Valley in 1856, it suggested that Neanderthals became extinct without contributing to the modern human gene pool (Krings et al. 1997). Over the last 20 years, developments in ancient DNA techniques have revolutionized, making it possible to reconstruct a nuclear genome, not only of Neanderthals (Green et al. 2010), but also of another extinct species of archaic humans, the Denisovans, who were identified as a distinct human species without a fossil record, solely from DNA extracted from a finger bone (Krause et al. 2010; D. Reich et al. 2010). When the nuclear Neanderthal genome was sequenced, it suggested gene flow from Neanderthals into modern humans: 1–4% of the genomes of people outside Africa are derived from Neanderthals (Green et al. 2010). This implied that, rather than becoming extinct, part of their DNA lives on in people today.

By providing enormous amounts of genomics data, human paleogenomics is said to have the potential to settle long-lasting debates that originated from the incompleteness of the archaeological and paleontological record (Lalueza-Fox and Gilbert 2011). Molecular genetics provided a new and genome-oriented way to understand what makes humans unique (Pääbo 2014a). Comparing the genome of Neanderthals with that of present-day humans was expected to provide us with a high-resolution picture of ourselves, thereby enabling us to identify what made us fully human.

The work of the Swedish geneticist Svante Pääbo and his team provides a remarkable case history in this respect. In his book *Neanderthal man: in search of lost genomes* Pääbo explains how he set out to retrieve the Neanderthal genome in order to find the genetic differences between them and present-day humans. His book begins with a eureka-like shout of joy, resounding in the ‘ancient DNA laboratory’ at the Zoological Institute of the University of Munich in 1996, namely: ‘It’s not human’! indicating that, apparently, Neanderthal DNA is interesting *because and insofar as* it diverges from ‘our’ genetic code. From an oblique perspective, Pääbo’s narrative is particularly fascinating as it involves a researcher who persistently tried to uncover the genetic underpinnings of that what is unique to humans: the genetic factor X that explains the series of cultural and technological explosions that eventually allowed ‘us’ to dominate much of the planetary biosphere. Bone fragments become depositories of paleo-DNA and DNA sequencing machines are employed to produce strings of A’s, C’s, G’s and T’s, shorthand for the molecular structure of DNA. With the help of such machines, Pääbo hoped to trace the genes that distinguish ‘us’ from ‘them’. Or, as Pääbo himself phrases it:

Among the few differences one would expect to find in the Neanderthal genome, there must be those that set us apart … Those few differences must form the biological foundations of the radically new direction our lineage took with the emergence of modern humans: the advent of rapidly developing technology, of art in the form we today immediately recognise as art, and maybe of language and culture as we now know it. If we could study Neanderthal DNA, all this would be within our grasp (p. 4).

The research explicitly started from the supposition that Neanderthals are ‘profoundly different from us’ (p. 14) and that Neanderthal DNA is ‘very different from the DNA of modern humans’. The expectation was that powerful DNA sequencing machines and PCR technology would now provide a high-resolution window into the past (p. 42), so that the difference between them and us could now be fleshed out in terms of a limited set of decisive genes that exemplify the Neanderthal–*Homo sapiens* divide. As Pääbo phrases it:

‘Studying how we differ genetically from our closest relatives would potentially allow us to find out what changes set apart the ancestors of present-day humans from all other organisms on the planet. In essence, we would be studying perhaps the most fundamental part of human history – the biological origin of fully modern humans, the direct ancestors of all people alive today (p. 72).

His research consistently revolves around the question of ‘what makes humans unique … what had set humans on an evolutionary track so different?’ (p. 83). The published version of the Neanderthal genome (in *Science*) was expected ‘to give us a picture of ourselves’ so that we can see ‘the essential genetic changes that make us human—the things that made our emergence as a global species possible’ (p. 220). Yet, interestingly, while Pääbo’s descriptions become increasingly technical, it becomes increasingly difficult to link the project’s findings with concrete and allegedly unique aspects of human behaviour, experience or culture. Unintentionally perhaps, rather than enabling us ‘to directly identify the genetic underpinnings of the differences between Neanderthals and modern humans’, the research outcomes rather increasingly convey the message of how similar we are. Neanderthals shared, for example, the FOXP2 language gene with modern humans. Symptomatic perhaps is Pääbo’s persistent fear of ‘contamination’ already mentioned above: the possibility that, instead of Neanderthal DNA, researchers are actually sequencing modern human DNA (perhaps even their own DNA) which somehow contaminated ancient Neanderthal bones, for instance via dust particles in the lab where living human researchers work. Pääbo describes how a significant number of Neanderthal publications are indeed ‘contaminated’ by human genetic material: the claimed results are tainted as the isolation between living human bodies and Neanderthal bones proves difficult to maintain, so that DNA passes from the one to the other and blurs the difference. And this not only applies to Neanderthal research. Most of the DNA allegedly retrieved from iceman Ötzi likewise came from the several contemporary humans who handled his remains. In Pääbo’s own terms: fear of contamination (DNA traffic) between ‘us’ and ‘them’ inside laboratories became more than just a concern, it evolved into ‘paranoia’ (p. 9, p. 87), it became a real ‘obsession’ (Pääbo p. 9). But even clean results increasingly suggest continuity rather than discontinuity (similarity rather than dissimilarity) between them and us. Indeed, the most important and tangible result of Pääbo’s research so far, perhaps, is that Neanderthals genetically contributed to modern human DNA via interbreeding. Eventually, Pääbo contends that even a complete catalogue of all the genetic differences between Neanderthals and modern humans would not by itself tell us what *the* difference between Neanderthals and modern humans would be (p. 226). Thus, the end result of Neanderthal genomics seems to be that, after exposure to the data deluge of next-generation sequencing technologies, the portrait of Neanderthal humans becomes increasingly—*human*.

In more recent publications, Pääbo recommends against imagining separate species within human evolution. It is not a question of an ‘us’ and a ‘them’, he now argues, but of one hominin meta-population. At the same time, he persistently contends that ‘one population in this meta-population—modern humans—ended up being very special, spreading across the world and, by force or competition, caused every other hominin group to become extinct.’ The key question is, according to Pääbo, what biological features made this population (made ‘us’) special? (Pääbo 2015). Although genomic differences appear not to be very numerous, this might change when our ability to identify functional variants in the genome improves (Pääbo 2014b).

## Darwinian winners and losers

Although paleogenomics was expected to have the potential to settle the debate and provide us with answers to the question who we are and what made us special, it increasingly revealed continuity and similarity between them and us. And yet, most Neanderthal researchers still seem bent on discovering minimal differences, based on the assumption that *because* ‘we’ survived and ‘they’ became extinct, our ancestors must have been superior to them somehow.

In 2014, for instance, Jamie Shreeve published an interview with Neanderthal expert Chris Stringer (Natural History Museum in London) in *National Geographic* under the telling headline ‘Gap between Neanderthals and us narrows, but does not close’ (Shreeve 2014). In his introduction, Shreeve points out that Neanderthals, notwithstanding the fact that they had big brains, made fairly complex stone tools, and expanded into Asia for nearly 300,000 years, became extinct in the end, while ‘we’ took over the planet. For that reason, scientists for decades have tried to pinpoint what particular ‘inadequacy’ led to the Neanderthal’s demise, and what ‘special property’ made us fully human and them not quite. Stringer replies that, although in earlier books he had taken the view that there was a major behavioural gap between the Neanderthals and us, he now agrees that recent evidence has indeed considerably ‘narrowed that gap’. Still, Stringer argues that this gap has not completely closed. Although they had speech and language, it was ‘a language for the here and now’, a practical language, fit for mere survival, but unfit for expressing complicated messages and the kind of hypothetical reasoning that leads to modern inventions. The emergence of complex language, giving modern humans a crucial fitness advantage, is a common explanation for the demise of the Neanderthal (Villa and Roebroeks 2014). It has been suggested that Neanderthals had a more restricted language system because of, for example, a shorter childhood (Langley et al. 2020) or a restricted working memory (Rossano 2010).

Twenty-five centuries ago, Greek philosophers such as Plato and Aristotle already considered mind or reason as the defining characteristic of human beings, and the disappearance of Neanderthals and evolutionary success of *Homo sapiens* is often explained by brain differences. Wynn and Coolidge, for example, argued that a ‘relatively simple’ genetic mutation about 100,000 years ago led to enhanced working memory capacity. This was allegedly the final evolutionary development that modernized the human mind (Wynn and Coolidge 2007, 2011). Although Neanderthals were very good at deploying learned quasi-automatic responses, their more restricted working memory placed limits on their ability to solve new tasks. As Neanderthals ‘lacked the inventiveness, characteristic of people today’, they became extinct while *Homo sapiens* prospered (Wynn and Coolidge 2008).

Pearce et al. (2013) argue that there is a difference in brain organization between Neanderthals and anatomically modern humans. A comparative study on endocast volumes suggests that Neanderthals enlarged their visual and somatic regions, with the retention of physical robusticity, whereas modern humans concentrated neural investment in social cognition. While the physical response to fluctuating conditions adopted by Neanderthals may have been very effective at first, the social response developed by anatomically modern humans seems to have won out in the face of climatic instability that both species experienced. Recently, morphological differences in the brain were investigated using 3D reconstruction of the Neanderthal brain based on computational neuroanatomy. The reconstructed average brains suggested that Neanderthals had smaller cerebellar hemispheres than Homo sapiens, particularly on the right side. These neuroanatomical differences may have caused differences in cognitive flexibility, language processing and memory capacity between the two species, researchers argue, and might have contributed to the replacement of Neanderthals by early *Homo sapiens* (Kochiyama et al. 2018). Although the evidence provided by these research efforts is inconclusive, they are interesting from an oblique perspective precisely because they are guided by the logic of hyperseparation. There *must* be a decisive difference, however small and subtle, and the research is geared towards recovering that factor X which put us on the track towards becoming a global species of ‘winners’, while Neanderthals (as ‘losers’) went extinct.

Geneticist Alysson Muotri and his team take this even one step further. Unlike skulls, brains do not fossilize, and therefore they are now trying to literally ‘recreate Neanderthal minds’, using the genome-editing technique CRISPR-Cas9. Human stem cells are engineered so as to include Neanderthal genes and grown into cortical ‘minibrains’ that should reflect the influence of ancient DNA. Compared with modern human minibrains, the Neanderthal organoids make fewer synaptic connections, these researchers argue, which may have influenced their ability to socialize, again a minimal but allegedly decisive difference which may explain their demise (Cohen 2018). Muotri states that we are very different from Neanderthals, whose brains were limited in their ability to create technology, art, imagination and overall culture. He sees our social brain as one of the key distinguishing factors between humans and other primates and is currently investigating what properties were likely responsible for limiting the Neanderthal’s social, cultural, and technological development and contributed to their extinction (UC San Diego School of Medicine 2018). Again, we would argue that the logic of hyperseparation is definitely at work here. There *must* be a difference between them and us, some genetic or neurological cause which explains our remarkable success, and this conviction is explicitly guiding the research. Overall, whereas the actual differences are become increasingly minimal and technical, it is clear that *the difference* between them and us is what these researchers are persistently after: a particular *signature* feature which may offer an explanation of why *they* became extinct while *we* (the ‘favoured race’, to use the Darwinian phrase) survived.

Other researchers have been looking for differences within the framework of dietary ecology. Several authors have argued that Neanderthals were unable to acquire as many calories from the Pleistocene environment as we modern humans could. It has been suggested, for example, that Neanderthals focused primarily on large prey and were less capable of switching to smaller animals like rabbits, compared to modern human. When conditions changed, and large game disappeared, Neanderthals were easily outcompeted by the more generalist modern humans, these authors argue (Fa et al. 2013). However, other evidence suggests that Neanderthals dietary regimes were actually quite comparable to those of modern humans, and included, among others, marine resources and plant foods (Henry et al. 2011, 2014), and recently, molecular texture analysis has even reversed the picture. Neanderthals were shown to alter their diets in response to changing local environment, exploiting resources only when they were more abundant and easily accessible, while modern humans on the other hand seemed to have maintained a stable diet despite fluxes in their local environment. Yet, in accordance with the logic that the de facto replacement of Neanderthals by modern humans suggests that the latter *must* have had some advantage over the former, it was now argued that modern humans were able to free themselves from environmental constraints, probably with the aid of superior technology, so that a more efficient and flexible exploitation of dietary resources might have given modern humans an advantage over Neanderthals (El Zaatari et al. 2016; Henry et al. 2014). In other words, different and sometimes contradictory argumentation strategies are deployed in order to reach a predictable conclusion: regardless of whether our diet was more flexible (responsive to environmental fluctuations) or more stable (autonomous), ‘our’ performance excelled in the end. Other researchers argue that if foraging adaptability cannot adequately explain a competitive disadvantage for Neanderthals, other factors must be considered, showing that differences in metabolic budgets, and less, or less effective, use of fire, inevitable lead to Neanderthal extinction (Goldfield et al. 2018). While according to Stewart et al. (2019) it was the hunting style that differed, and the endurance-based long distance running *Homo sapiens* outcompeted the muscular sprinter when the environment changed (Stewart 2019)

Currently, an important focus of the debate is whether Neanderthals were ‘conservative’ while modern humans were ‘innovative’. According to Pagagianni and Morse, for instance, Neanderthals showed ‘limited capacity for innovation’ (p. 89) because during the Pleistocene their use of stone tools and hunting methods hardly seemed to change, so that their ‘conservatism’ is considered ‘very strong’ (p. 97). Although experts now consider Neanderthals as far more advanced than previous generations of researchers did, their ability to innovate is still generally seen as limited (p. 99). And this seems linked to another advantage modern humans were beginning to develop around 50,000 years ago, namely the use of ornamentation, notably in the form of body paint (red ochre) and in the form of jewellery (using perforated beads and shells, p. 106), thereby perhaps exploring new forms of symbolic expression. Was this the key to their (to ‘our’) success: new forms of symbolism as a decisive milestone (p. 120)? However, this seems to suggest that the divergence between modern humans and Neanderthals was a cultural event rather than something in their genes. The general idea is that, whereas Neanderthals were living in the here and now, new symbolical codes may have opened up a much more extended ambiance of culture for modern humans, but on closer inspection the evidence again seems indecisive. According to some researchers, Neanderthals also used red ochre, albeit sporadically, perhaps even as a form of symbolism. It seems indisputable by now that they used eagle talons for ornamentation (p. 120). And indeed, the Neanderthal cross-hatch (‘hashtag’) pattern in Gorham’s Cave, Gibraltar, may also point to incipient symbolic thinking, although Papagianni and Morse hasten to add that Neanderthal symbolism ‘was not nearly as extensive as the modern human use of shell jewellery’ (p. 120).

The approach adopted in these more recent reviews is already much more nuanced than the argumentative strategies in previous summaries of Neanderthal research. For instance, in their book *In search of Neanderthals, solving the puzzle of human origins*, Stringer and Gamble (1993) still doubted that Neanderthals had intelligence and memory at all and claimed that Neanderthals depended on biological rather than on cultural solutions for survival. Yes, Neanderthal burials may seem symbolic, but they probably lacked a genuine symbolic rationale, Stringer and Gamble argued. Rather, these rituals were limited repetitious behavioural forms, resulting from mere imitation. And yes, Neanderthals could plan, but only with limited depth compared to modern humans. The structures they created at Molodava and Arcy-sur-Cure resembled ‘nests’ rather than symbolic human ‘homes’. Their society was not as complicated, and this explains, among other things, why they could not travel over sea. Therefore, they remained Old World hominids. Indeed, they may have imitated certain aspects of modern human behaviour, but they could not fully understand it.

Similar discussions can be encountered in the book *The singing Neanderthals* by Steven Mithen (2006), who looks at Neanderthal research from a slightly different angle, focusing on the relationship between early human language development and music. Building on contemporary Neanderthal research, Mithen argues that Neanderthals developed a holistic (i.e. non-compositional) proto-language, which he refers to as *Hmmmm*-communication, giving rise to a nonverbal, pre-linguistic, musical mode of thought. Because Neanderthal culture was characterized by stability and stasis, Mithen argues, they did not need a compositional language (which would have allowed them to exchange complex messages). They communicated about the here and now. Absence of innovation meant that they could rely on tried and tested methods so that their language was holistic, consisting of fixed utterances. *Homo sapiens* utterances, Mithen argues, were different, namely compositional and referential, which means that phrases could be broken into separate units of meaning which could be recombined to broaden the communicative repertoire. Because of their ‘immense cultural stability’, Mithen argues, Neanderthals did not have much to say, which explains the absence (or marginal presence) of symbolical objects. This is exemplified by their ochre use. While black ochre was used by Neanderthals, probably to protect their skin (p. 252), the use of red ochre counts as an early *Homo sapiens* innovation (p. 30; p. 253), basically because the colour red (used as body paint, to highlight certain body parts for instance, such as faces and breasts) is associated with symbolic activity (for instance: indicating allegiance to a particular clan). Red ochre may be regarded as the first symbol to be used by humans (p. 253). Although some of Mithen’s descriptions are quite enticing, the problem is that he persistently clings to a dichotomous or binary way of thinking (them versus us, holistic versus compositional, black ochre versus red ochre, etc.). The logic of hyperseparation is still at work, albeit now focusing on cultural rather than genetic differences. Instead of starting from the Neanderthals = holistic and modern humans = compositional dichotomy, why not see the shift from holistic to compositional as a gradual pattern of evolution of language *as such*, which may have affected *both* Neanderthals and modern humans, and which is still ongoing by the way (because remnants of holistic language are still in vogue in human language even today)? If this perspective is taken, the tension between holistic versus compositional becomes a matter of circumstances and socio-cultural ambiance, rather than reflecting the absence or presence of certain (gene-based) properties, associated with certain (typically human?) ‘survival’ genes.

In his book *The humans who went extinct: Why Neanderthals died out and we survived*, Clive Finlayson (2009) starts from the position that Neanderthals had parallel and comparable minds, and were our equals in brain power and cognitive abilities. Their disappearance was a matter of changing circumstances, in other words bad luck. Yes, the hashtag at Gorham’s Cave was truly a revelation for those who saw the Neanderthals as dumb and incapable brutes that somehow survived for over a quarter-of-a-million years on the Planet. Still, Finlayson maintains that modern humans were decidedly more innovative. ‘Throughout human evolution populations that could not handle a rapid rate of change in their environment caused by some perturbation went extinct. The Neanderthals are a prime example.’ (p. 219) If one thing makes us unique, he argues, it is our ability to change things if we choose to.

Ian Tattersall starts from the opposite position, considering modern humans uniquely gifted creatures, distinctly different, unprecedented, extraordinary, and overwhelming all competition whilst taking over the world. In his book *Masters of the planet* (Tattersall 2012), he argues that language and technology are what define us as modern humans. Our aptitude for symbolic reasoning and our unique mode of processing information mark us off as different from other creatures, so that there is a deep cognitive gulf separating us from other living organisms. Neanderthals are exceptional in that they are so similar to us in many ways, and yet the gap remains. Yes, Neanderthals knew practices of burial, but it is less probable that they believed in an afterlife: something that would demand symbolic cognitive abilities. Yes, their Mousterian toolkit was intelligent and dexterous, but at the same time extremely monotone: there was no experimentation with different ways of doing things, as modern people do: Neanderthal craftsmanship was ‘stereotyped’. Yes, they knew language, but nothing in their technological record suggests that they were symbolic thinkers. Yes, they were skilful and complex, but not in the way *we* are. Neanderthals were not innovative: there was never a qualitative break with the past. We are different, we are symbolic. Neanderthals relied on the ‘old-style’ hominid way of dealing with the world, relying on intuitive processes, but ‘we’ (symbolic modern humans) began to process information in an entirely revolutionary and unprecedented way. At the same time, Tattersall agrees that it is unclear how this difference can be explained. For him, it is not a question of better genes or better brains, but of a cultural event: humankind’s symbolic awakening.

In *The Invader, how humans and their dogs drove Neanderthals to extinction* (Shipman 2015) taphonomist and science author Pat Shipman describes modern humans as ‘a supremely well-adapted invasive species’ (p. 8). She provocatively claims (admitting that genetic evidence is yet to be found) that the deciding factor in the success of our ancestors was the domestication of wolves. This ability to capture and domesticate wolves was either unknown to Neanderthals or beyond their capabilities. Given the stability in Neanderthal diets and tools, she claims, it appears they were slow to innovate and slow to change their ways. Our ability to enlist other species and use them to enhance our own survival, in combination with climate change, explains Neanderthal extinction and modern human success.

## Assessment: the minimal difference and the modern human superiority complex

We argued that Neanderthal discourse is a vantage point from which we may recognize, and reconsider, the anthropocentric logic at work in the formation of different kind of oppositions and hierarchies when it comes to understanding ourselves as humans. By studying the Neanderthal discourse from an oblique perspective, we attempted to show how our understanding of Neanderthals is affected by the desire to safeguard human uniqueness, genetically, but also behaviourally and culturally. The overall trend, we noticed, is to identify the human mark or spark, which defines us as favoured ‘winners’. The documents consulted above indicate that, although the gap between Neanderthals and modern humans continues to shrink, scientists at the same time continue to look for an increasingly minimal difference, something subtle but crucial, separating them from us, although the actual differences presented to explain the gap all prove open to dispute. Instead of focusing on the question where to draw the line between us and them, on pinpointing that signifying difference, we would like to shift the focus to the question why we persist in drawing a line in the first place. Why this emphasis on difference and separation, why not think about Neanderthals in terms of familiarity and continuity, of diversity and multiplicity?

Seen from an oblique perspective it is interesting that, like the Human Genome Project itself, Neanderthal genomics and related forms of palaeoanthropology resulted in a narcissistic offence (Zwart 2007). Whereas it was expected that his type of research would answer the question who we are, would reveal the ‘factor X’, the genetic predisposition that sets us apart from other hominins, the (inevitable) outcome was that these differences become increasingly minimal and eventually evaporate. And yet, although the difference is diminishing, most Neanderthal experts continue their quest for the minimal difference that somehow must be there, shifting their focus from genetics to behaviour and culture and back. The conviction that there must be a difference between them and us, and that we expect palaeoanthropology and paleogenomics to inform us what this difference exactly is, has been a basic philosopheme, a basic conviction guiding this area of research, implicitly or explicitly. Although one ‘marker of humanness’ after another had to be abandoned or redefined, there is a strong tendency at work in this research practice to keep looking for that essential difference, that unique feature, that defining characteristic of humanity, the miraculous spark that made us radically different.

This focus on difference affects both the *context of discovery* and the *context of justification* of Neanderthal research. First of all, as we described above, the context of discovery of Neanderthal research tends to be focussed on discovering difference from the very outset. The work of Svante Pääbo concerning the Neanderthal genome, for instance, was explicitly designed to reveal the genetic differences ‘that set us apart’ and that allowed ‘us’ to take a radical new evolutionary direction. A similar focus serves as point of orientation for the work of Neanderthal experts like Chris Stringer, Alysson Muotri and others. As indicated above, it serves as a key motivation for their research. But the focus on difference is recognisable in the context of justification as well, notably in the recurrent argument that, if the expected difference is not confirmed by evidence, we must proceed to look for it elsewhere, so that the focus is *displaced*, towards ever more subtle differences, rather than abandoned.

Building on Plumwood’s conceptual framework, we have argued that this focus on difference, on discontinuity rather than continuity, is connected with the dualist, hyperseparated conception of the human. Our concept of the human is based on a tenacious set of assumptions about the inferior status of the nonhuman world. What is taken to be authentically and characteristically human is not to be found in the nonhuman, so that we see ourselves as separate and distinct. A dualistically construed dichotomy typically polarizes difference and minimizes shared characteristics. Moreover, it construes difference along the lines of superiority/inferiority, and views the inferior side not as an end in itself, but as instrumental to the higher or superior side (Plumwood 1991). By emphasising the significance of distinguishing characteristics, even if shared qualities are abundantly available, separation between the dualized spheres is safeguarded. Ultimately, the focus is not on what we have in common, but on what the other lacks. In other words, as long as we look at Neanderthals from a perspective of hyperseparation, every allegedly unique human feature will be highlighted with a magnifying glass. In order to guarantee distinctness, there simply *has* to be a defining (minimal) characteristic which singles us out as different. As a rule, characteristics that are seen as essential to defining the upper side are used to define the other (inferior) side as well, but now in the sense that they lack these characteristics. Innovation, for instance, may be used to define Neanderthals, but in a negative manner, as something which they allegedly missed. With the help of characteristics such as ‘being innovative’, the familiarity and closeness of Neanderthals is negated. The other is relegated to a position of oppositional subordination and is not considered as an independent other, with their own needs, identity and aspirations. The qualities of the other are either negative features (deficiencies), or the result of failings, while qualities that do not fit into the scheme tend to be ignored or explained away.

Hyperseparation is a key indicator of dualism, of thinking in binary terms. It creates a questionable dichotomy, however, treating the opposite pairs as two distinct spheres of existence between which a gap, a rupture unfolds. It creates an illusion of separation, making it literally unthinkable to recognise the other as *both* alike and different. It obfuscates the continuity and ‘familiarity’ between them and us. Neanderthals, familiar and strange at the same time, incite in us an uncanny feeling. While we regard them as basically human, we still try to keep a distance and use them to define our own self-image, often based on series of oppositions such as sluggish versus agile, brutish versus smart, conservative versus innovative, etc. Thus, the Neanderthal story emerges as a tale about insiders and outsiders, exploring innovators and conservative savages, reflecting the desire to see ourselves as different somehow, not only from other mammals, but from pre-modern humans as well (Cartmill 2012). An ‘uncanny valley’ (Morton 2018) separates them from us and keeps them at a distance, even if, due to paleoanthropological research, they are coming increasingly close.

Not all Neanderthal experts, however, fall into this trap. The pervasive use of double standards—considering the in-group, or upper side, capable until proven incapable, while the out-group is incapable until proven capable—in the assessment of Neanderthals has been pointed out and questioned by a number of Neanderthal researchers themselves (Roebroeks and Corbey 2001; Speth 2004; Villa and Roebroeks 2014). In their 2014 PLoS paper entitled ‘Neandertal Demise: An Archaeological Analysis of the Modern Human Superiority Complex’ (Villa and Roebroeks 2014), Paola Villa and Wil Roebroeks reviewed the archaeological evidence for a series of claims which all build on dichotomies that suggest that early modern human *had* something which Neanderthals lacked. First of all, anatomically modern humans (AMH) allegedly had a complex and syntactical symbolical language, while Neanderthals did not. Moreover, while AMH were innovators, the capacity of Neanderthals for innovation was much more limited. AMH used projective technology, while Neanderthals did not. Modern humans had a diverse diet, but Neanderthals a restricted one. AMH used traps and snares, while Neanderthals did not. AMH had extensive social networks, while Neanderthals did not. Last but not least: while AMH hafting techniques entailed complex procedures requiring abstract reasoning and ‘modern cognition’, hafting of tools by Neanderthals was a simple procedure, only using naturally available glues. Yet, Villa and Roebroeks systematically show that for all these contentions the evidence is inconclusive. They conclude that the available data do not convincingly support the supposed technological, social and cognitive inferiority of Neanderthals compared to their AMH contemporaries. Characterizations of Neanderthals as cognitively inferior to modern humans, they argue, relies on a framing which overemphasizes the significance of subtle differences.

Paleoanthropologist João Zilhão takes a similar position and argues that, depending on different perceptions of the behavioural basis for the triumphant status of civilized society, Neanderthals are represented as lacking the corresponding behavioural feature (Zilhão 2011). According to Zilhão, present-day palaeoanthropology is ‘infected’ with progressivist views, so that the need to place ‘us’ at the top of the ladder of life still prevails. Modern humans see themselves as the masters of evolution, and we keep looking for what we are not (or not anymore). Double standards are especially problematic when studying cognition, because cognition as such does not fossilize and we have to rely on indirect inferences. We study Neanderthal cognition using modern human cognition as a reference frame, which is problematic in itself. In Neanderthal research, a *scala naturae* view of cognition still seems at work, so that the issue of Neanderthal cognition is redefined as the question of what fraction of our abilities they possessed (Johansson 2014). The branching tree may have replaced the ladder as the modern biological model of evolution, but also the branching tree may be a deceptive metaphor, as DNA evidence reveals a network of connections, described by paleoanthropologist John Hawks as a ‘muddy river delta’ (Hawks 2016), rather than a tree. When it comes to models of cognitive and behavioural evolution, the dominant narrative still typically implies a linear approach, with the implicit assumption that the ‘behaviourally modern way’ is by definition the cognitively most sophisticated way (Langbroek 2012).

As indicated, paleogenomics was expected to settle long-lasting debates that originate from the incompleteness of the record produced by the traditional tools of archaeology. Paleogenomics could take us beyond the impasse by reframing the debate and breaking down barriers. With the power of hard data, stereotyped expectations would be shattered, and new windows would be opened up (Reich 2018). But this optimistic view separates the researchers from their technology. Their object of study becomes a digital resource generated by genomics (M’charek 2005). Yet, hard data, established with the help of high-tech equipment, will not produce a coherent and convincing picture of Neanderthal life all by itself. Quite the contrary, interpretation (by researchers) continues to play a decisive role. The picture of Neanderthal existence that is based on digitalised genomics data alone is bound to remain technical and lifeless, abstract and fragmented, so that researchers will continue to use their (evidence-based) expectations and imaginations to fill in the gaps, for instance when it comes to developing plausible interpretations of ambiguous findings or formulating hypotheses for future research. Geneticists are often motivated by the hope, or conviction, that genetics will provide insights into the biological nature of how humans differ from other species. They are searching for that crucial difference that makes us uniquely human. As a result, although boundaries are re-established, the conceptual framework, the activity of inclusion and exclusion that is embedded in the routines of genetics, remains intact. In comparisons, the other consistently tends to fall short if assessed on the basis of certain standards which allegedly indicate what is to be considered truly and normally human.

The obsession with (minimal) differences is symptomatic for it reveals the desire to impose clear demarcations when it comes to upholding our identity. In the light of scientific evidence, continuity rather than discontinuity appears to be the rule, and it has become increasingly difficult to explain how our uniqueness can be upheld. When Neanderthals are regarded more and more like us, it abolishes their ‘otherness’. And to the extent that binary separations evaporate, the question of who we are, the question of humanity, becomes increasingly uncertain. Although researchers develop technological windows that provide access to the lost world of Pleistocene existence, Neanderthals continue to serve as mirrors reflecting how *we* want to see ourselves. Interpretations of fossil specimens have always been influenced by assumptions about what is to be considered ‘human’ or ‘humanness’ (Corbey 2012; Lequin 2018). Neanderthal discourse is still under the sway of tenacious implicit biases and preconceptions, and this notably applies to the widespread conviction that, *because* ‘we’ survived and ‘they’ became extinct, our ancestors must have been superior to them. Many studies that assign a major role to a selective advantage of modern humans in the Neanderthal’s demise, do so based on the premise that such an advantage had to exist in order to explain Neanderthal extinction, and they focus on determining exactly what this selective advantage could have been. This assumption might be unnecessary and evidence to support its claims should be interpreted cautiously (Kolodny and Feldman 2017). It presupposes a normative, social Darwinist conception of evolution, explaining extinction and survival on the basis of inherent biological superiority or inferiority. It suggests winning and losing is natural and inevitable and forges an image of a clear dichotomy between the groups involved (O’Brien and Leichenko 2003).

Moreover, although views and theories about Neanderthal existence are informed by quickly expanding collections of findings, they remain sensitive to the broader socio-cultural environment. One could argue, for instance, that the current focus in Neanderthal research on innovation as a typical human feature (so that ‘we’ are ‘innovative’ while ‘they’ are ‘conservative’) basically reflects the current neo-liberal zeitgeist, rather than being an objective paleoanthropological statement of fact. We define ourselves as innovative because in contemporary dominant culture, innovation is considered something positive. The innovation theorem should raise suspicion precisely because it is in accordance with the syllogism: We were better at something; traditionalism is ‘bad’ and innovation ‘good’; ergo, we were more innovative.

## Conclusion: dismantling dualism

Human-centeredness is not serving us, nor others, well. The way to proceed, we argue, is to dismantle the deep dualism that underpins our superiority complex. This would be beneficial not only for Neanderthal research, but also for contemporary human culture on a more general plane. What if we stop trying to fit every new piece of evidence into this rating system that essentially presupposes that we were more competitive, and therefore superior? What keeps us from reading the Neanderthal evidence in a different manner? The alternative is not pure homogeneity, equality without any diversity, however. Neanderthal existence may have been different, but also similar, and it is intriguing to use technoscientific tools to try to enter their world (technoscientific time-travelling, if you like). The problem is perceiving diversity in terms of dichotomy and hierarchy, rather than in terms of diversity. Dismantling dualism does not imply erasing all differences, nor reversing the value of the poles (seeing Neanderthals as the Noble Savages of the genomics era and ourselves as estranged and degenerated). Instead, it requires recognition of a complex interacting pattern of both continuity and difference. It involves recognizing the relationship and continuity between self and other, but also affirming the independence of others, with their own ends and needs, alike and different at the same time (Plumwood 1993b). A non-hierarchical concept of difference will affirm continuity and reclaim the obfuscated areas of overlap.

Although Neanderthals are now generally regarded as basically human, it is often not that obvious what the starting point, the null hypothesis in Neanderthal research is. When studying animals, the null hypothesis usually is to assume that they are different and if we discern similarities, we opt for the simplest possible explanation to avoid anthropomorphism. In anthropological studies of humans, the opposite applies, namely the assumption is that others are people like us, and we take care not to interpret diversity in terms of difference. What applies to Neanderthals? Do we consider them fully human, or rather as somewhere on the way towards becoming human (Johansson 2014)? Neanderthal research generally seems to start from the assumption that they are different, a choice (often implicit) that colours the interpretations of the data and the conclusions of research. If we consider human and Neanderthal as basically equal, however, continuity becomes the null hypotheses. This implies an anthropological, intersubjective approach, so that paleoanthropologists become more similar to participant observers (Noske 1989), exploring human cultures, albeit of the past. To understand Neanderthals, we have to imagine what it would be like to be one of them, while at the same time being aware of the impossibility of complete identification and understanding. We should explore this lost Pleistocene world with respect, rather than with disdain.

As Plumwood argues, because of human-centeredness and the illusion of separateness, agency and autonomy, we run the risk of losing our ability to empathise with others. In order to develop a non-hierarchical concept of difference, or rather of diversity, we must thoroughly review the data, reconsider the traces of these familiar others, and reclaim a positive identity for them, one that is no longer based on inferiority, deficiency and lack (Plumwood 1993b). Here, Plumwood (2009) argues, we may learn from poetry and literature because poetry and literature have better methods to re-image the world in richer terms and delivering new perceptions, different stories with new characters and better plots. Fiction not only allows us to rethink the current worldview and give a voice to those who cannot speak for themselves, it can also reveal our implicit assumptions, and explicitly challenge our stereotypical and ideological self-images. Although science is often seen as the domain of facts, facts that will eventually put an end to fictitious beliefs, the distinction between fact and fiction is not that clear-cut, notably in paleoanthropology. Etymologically speaking, the term fact comes from *facere*, to produce. Paleoanthropological facts are produced, with the help of advanced equipment no doubt, but imagination always plays a role. Neanderthal research is a storytelling practice, comparable to primatology in many ways (Haraway 1989), but still a storytelling practice in its own right. The prevailing story is one of struggle and competition, of winners and losers. Our self-narrative aims to explain, but also to justify who we are, but in the current era of mass extinction and global ecological disruption, the concept of ‘winning’ has lost its positive connotation. We are in desperate need of new stories, about connectedness, rather than separation, in human culture in general, and in Neanderthal research in particular.

### Final comments

Although Neanderthal research has clearly progressed in terms of new discoveries and new techniques during its ‘golden years’ (Papagianni and Morse 2015), the implicit bias, the binary focus on difference in terms of ‘they’ versus ‘us’, providing ‘us’ with an evolutionary benefit, is still there. In this respect, we argue, the decisive step still has to be made. What we envision is not a research field ‘free from biases’, but one in which implicit biases are more readily acknowledged, considered and addressed. Distinctions between who gets to count as human and who does not, has created an ‘Uncanny Valley’ (Morton 2018), inhabited by ‘familiar strangers’. The nonhuman is separated from the human by an unbridgeable chasm. If we look out over the chasm at the definitely nonhumans at the other side, it is as if the chasm doesn’t exist. The valley, Morton argues, is an artefact of xenophobia, of fear of the other and of what we have in common with that other. Instead of recognizing that we are not that separate, nor that isolated, we try to keep the distance between our peak of distinction and the valley below, because the ambiguity of both familiar and strange provokes in us an uneasy, uncanny feeling. Emphasising difference implies pushing Neanderthals into this valley. Appreciating Neanderthal existence as a human lifeform, accepting and embracing their uneasy, strange familiarity, their ambiguity, means making the valley a bit shallower (Morton 2018).

The discovery of the partial skeleton in 1856 in the Neander Valley marked the beginning of palaeoanthropology as a distinct field of study. From the start, it has evolved around concepts of identity and inequality. Neanderthals are the longest known and best-known hominin species, but we now know that we have shared our world with many different human species during the last two million years or so. In 2015, a previously-unknown species of extinct hominin was discovered in South Africa (Berger et al. 2015). This hominin had a mosaic anatomy, with both primitive and humanlike features, and was named *Homo naledi*. In a book about this discovery, *Almost Human: The Astonishing Tale of Homo Naledi and the Discovery That Changed Our Human Story* (Berger and Hawks 2017) lead scientist Lee Berger describes:

For nearly a century, archaeologists had argued about whether Neanderthals recognized mortality, understood death, or buried remains of their dead. Neanderthals were fundamentally human, with a brain size and evidence for complex culture that rivalled those of modern humans. With *Homo naledi*, we were looking at a primitive creature with a brain only a third the size of a human brain today. Could it be possible that this species – clearly not human – still had the kind of awareness and social complexity that we see in our own species? (p.205)

It took a century for Neanderthals to be included as ‘fundamentally human’. Before them, around 1900, the Cro-Magnons of the Upper Palaeolithic, earlier interpreted as a kind of animal, crossed the threshold to humanness and were considered to be ‘people like us’, anatomically, behaviourally and cognitively (Corbey 2005). There is also considerable consensus about who is to be considered as clearly nonhuman. Nevertheless, merely expanding the circle of humanity, moving the boundary back in time, is not a particularly productive move. It means the quest for this signifying difference, some human speciality that makes us radically different, will continue. As long as the master model of humanity, based on exclusion of the inferior other, remains intact, the uncanny valley remains filled with many familiar strangers, like *Homo naledi*; ‘almost human’ and ‘clearly not human’ at the same time. The Neanderthal controversy is only the beginning.

## Footnotes

1 Any member of the taxonomic tribe Hominini. This includes recent humans (*Homo Sapiens*) together with extinct members of the human lineage, of the genus *Homo* (including *H. neanderthalensis*, *H. floresiensis*, *H. naledi, H. Luzonensis,* and the Denisovans), and more ancient forms like Australopithecines, *Paranthropus* and *Ardipithecus*.

2 In other words, Neanderthal research calls for a further extension and continuation of Haraway’s work: ‘Neanderthal Visions’. This paper aims to contribute to such a project.

3 Our geophysical impact as a planetary species on planet Earth has become omnipresent, irreversible and disruptive to such an extent that both geologists and philosophers have announced the birth of the Anthropocene as a new (καινός) and decidedly human (ἄνθρωπος) era, a moment of global crisis (Crutzen 2002; Hamilton et al. 2015; Steffen et al. 2011).

4 ‘On the Origin of Species by Means of Natural Selection, or the Preservation of Favoured Races in the Struggle for Life’ (Darwin 1859).
